# A Rare Case of Presacral Epidermoid Cyst in an Adult Male: Emphasis on Diffusion Weighted Magnetic Resonance Sequences in Preoperative Imaging

**DOI:** 10.7759/cureus.2050

**Published:** 2018-01-10

**Authors:** Muhammad I Alvi, Fatima Mubarak, Kumail Khandwala, Muhammad D Barakzai, Aisha Memon

**Affiliations:** 1 Department of Radiology, The Aga Khan University, Karachi; 2 Department of Pathology & Laboratory Medicine, The Aga Khan University, Karachi

**Keywords:** epidermoid cyst, presacral lesion, diffusion weighted sequences, magnetic resonance imaging

## Abstract

Epidermoid cyst of the presacral space is a rare congenital lesion of ectodermal origin. Presacral epidermoid cysts have been previously reported in women, however are extremely rare in males. We report a case of presacral epidermoid cyst in a 55-year-old male who presented to our emergency department with acute urinary retention and history of chronic constipation. A non-contrast computed tomography scan was performed with suspicion of urolithiasis, which revealed a well circumscribed low attenuation presacral mass. Magnetic resonance imaging (MRI) of the pelvis was subsequently performed to further characterize the lesion. The mass was returning hypointense T1 and hyperintense T2 signals with few foci of T2 hypointensities. There was no post-contrast enhancement; however the lesion was showing diffusion restriction, appearing hyperintense on diffusion weighted imaging (DWI) and hypointense on the corresponding apparent diffusion coefficient map. These imaging features were consistent with an epidermoid cyst. Laparotomy with complete surgical excision of the cyst and preservation of the adjacent structures was performed. The histopathology confirmed the diagnosis. This case highlights the importance of MRI with additional sequences of diffusion weighted imaging which can be helpful to differentiate, to a good degree of confidence, among different pelvic tumors, therefore obviating the need of biopsy before surgery.

## Introduction

Presacral epidermoid cyst is a rare congenital lesion of ectodermal origin. It develops from an ectodermal tissue remnant which is misplaced during embryogenesis due to faulty development of adjacent structures [1]. Presacral epidermoid cysts have generally been reported in asymptomatic women as incidental findings, but are an extremely rare occurrence in males [2]. Epidermoid cysts can be seen throughout the body, but are rare in retrorectal/presacral regions. The exact incidence of presacral epidermoids is unknown; however, a previous report suggests that they are responsible for one in 40,000 admissions [3]. The lesions can slowly grow over time and become infected and inflamed [4]. On histologic examination, these cysts have a thin wall lined by stratified squamous epithelium with a distinct granular cell layer, surrounding a mixture of desquamated debris, cholesterol, keratin, and water [1].

We present a case of a presacral epidermoid cyst in a male patient who presented with difficulty in passing urine and chronic constipation. The following case, to the best of our knowledge, is the third globally reported case of a presacral epidermoid cyst occurring in a male patient and an extremely unusual cause of chronic constipation and urinary retention [2, 5].

## Case presentation

**Figure 1:**
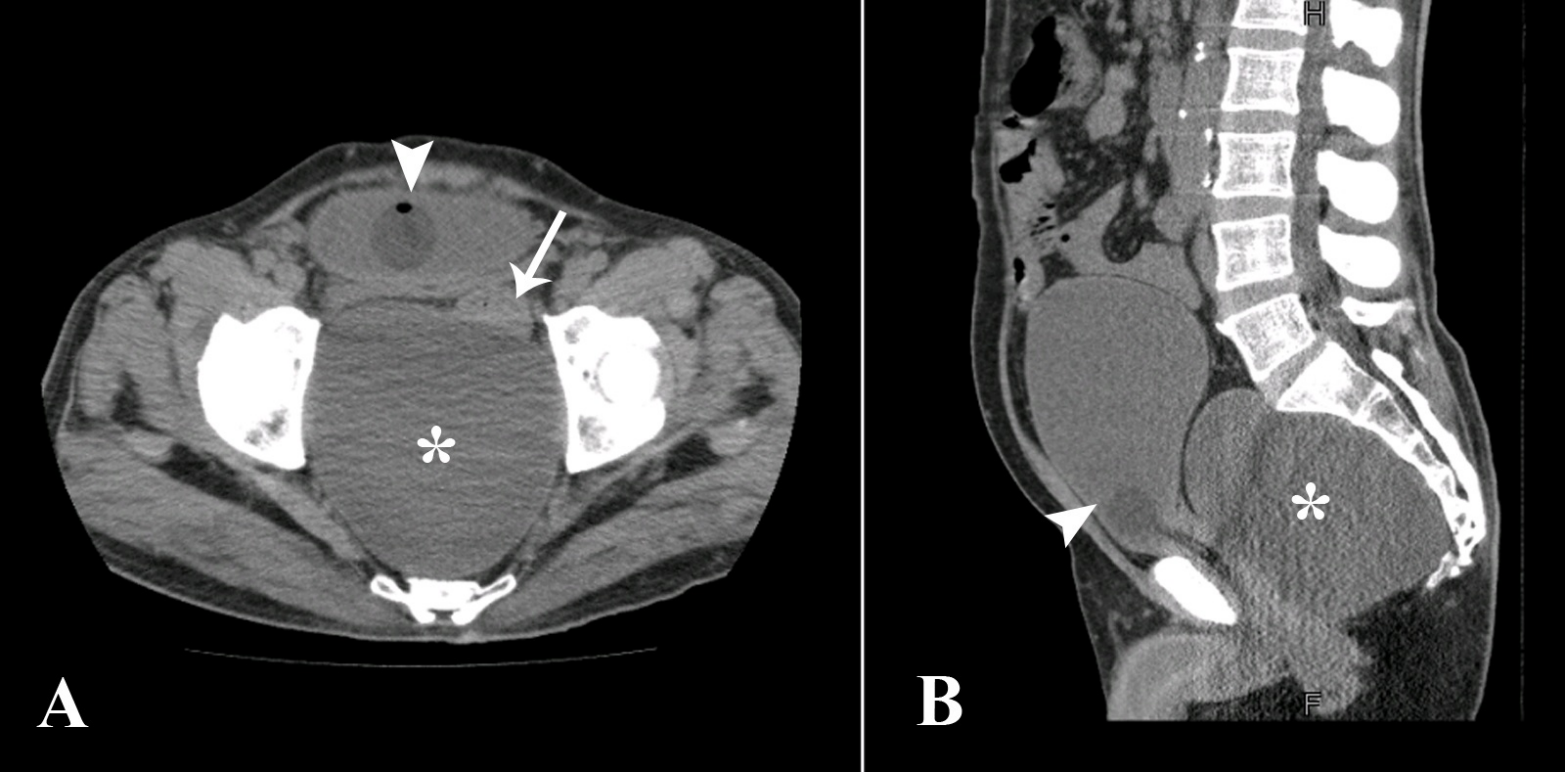
Axial (A) and sagittal (B) sections from CT-KUB A large well-defined low attenuation lesion in the presacral location (asterisk), displacing the urinary bladder superiorly and compressing the rectum in between (arrow). Foley’s catheter bulb can be seen in the bladder (arrowhead). CT-KUB: computed tomography scan of the kidneys, ureter and bladder

A 55-year-old male patient came to the emergency room (ER) with complaints of difficulty in passing urine for the past eight hours. He also gave history of chronic constipation on and off for the past ten years. A non-contrast computed tomography scan of the kidneys, ureter and bladder (CT-KUB) was performed on a multi-detector scanner which revealed a large well-defined low attenuation presacral lesion measuring 13 x 11 cm, which was displacing the rectum anteriorly and urinary bladder superiorly with compression over its base, resulting in pressure effects over these viscera (Figure 1).

**Figure 2:**
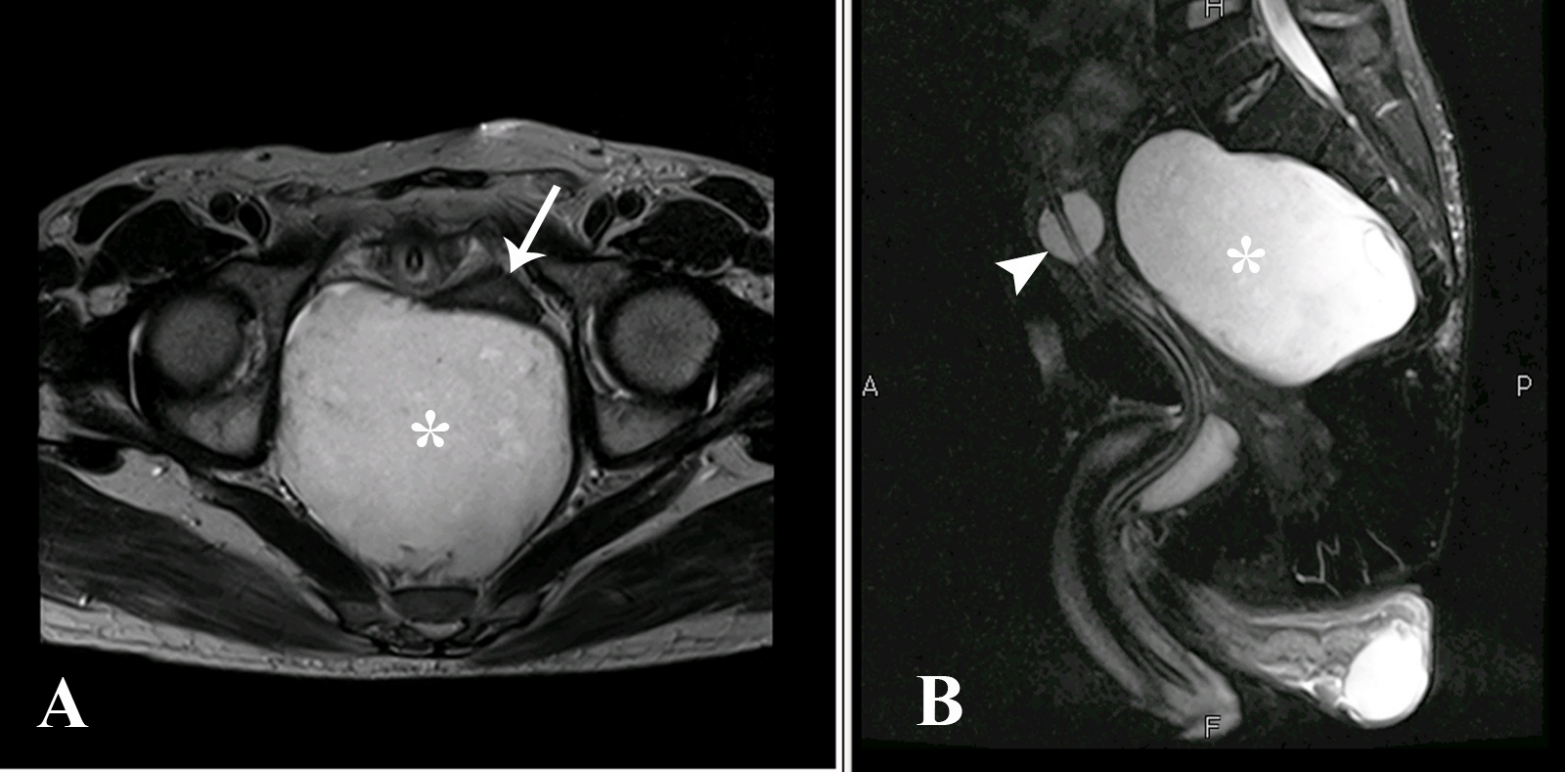
Axial (A) and sagittal (B) T2-weighted MR sequences The cystic lesion (asterisk) is hyperintense on T2 weighted imaging, with few foci of T2 hypointensities in the periphery correlating with keratin. It is compressing the rectum anteriorly (arrow) and the urinary bladder which contains a Foley's catheter in situ (arrowhead). MR: magnetic resonance

Subsequently, contrast enhanced magnetic resonance imaging (MRI) of the abdomen and pelvis with additional diffusion weighted sequences was performed on a 1.5-Tesla scanner for further characterization of the lesion. The scan revealed a well-defined mass in the presacral space returning hypointense T1 and hyperintense T2 signals with few foci of T2 hypointensities (Figure 2). No communication of the lesion with the spinal canal or bony involvement was noted.

**Figure 3:**
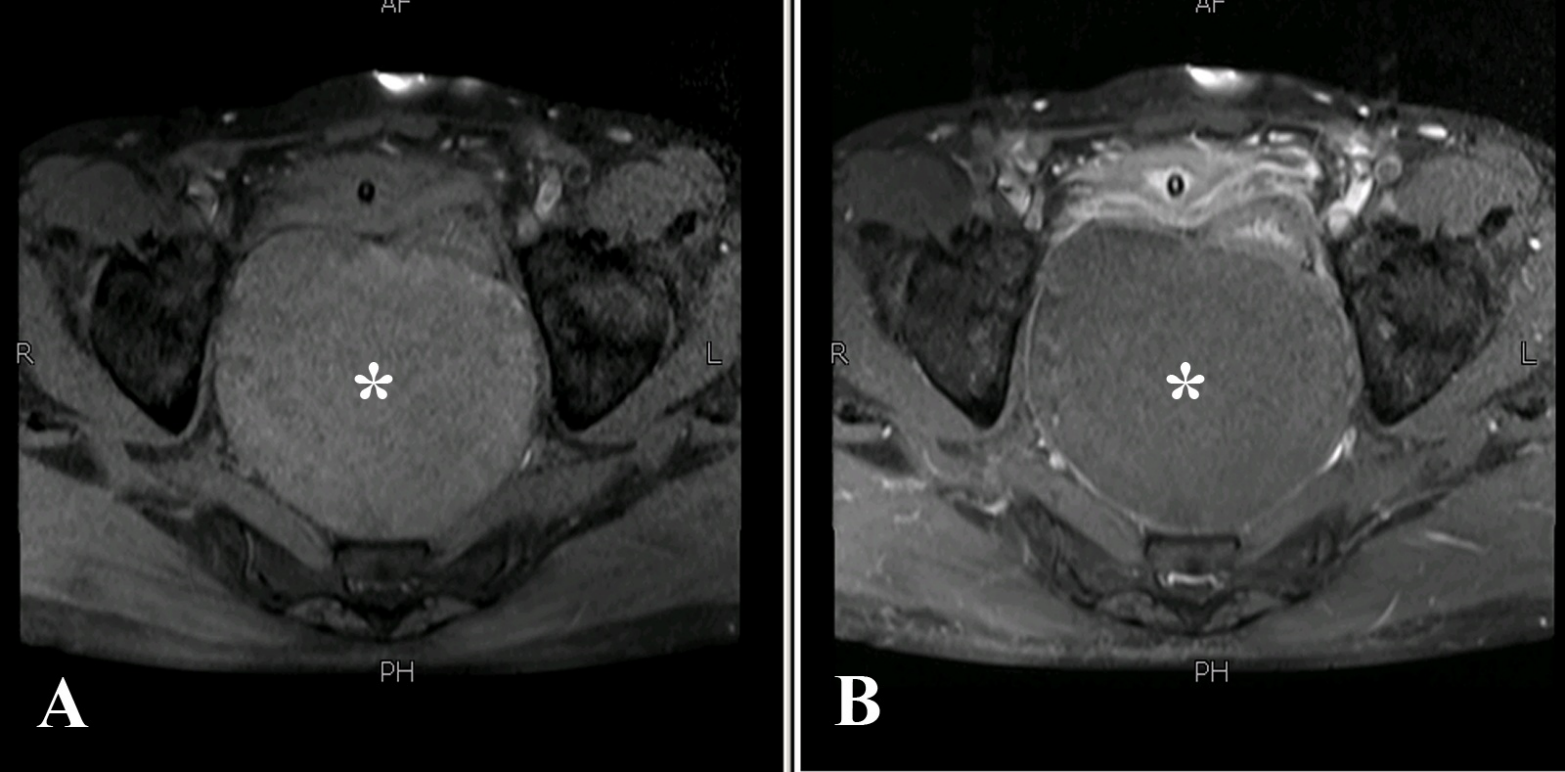
Axial T1 weighted pre (A) and post (B) contrast sequences The cyst (asterisk) demonstrates an extremely thin wall without any post contrast ring-enhancement or intramural solid nodule.

There was no post-contrast enhancement or enhancing solid nodule within the lesion (Figure 3).

**Figure 4:**
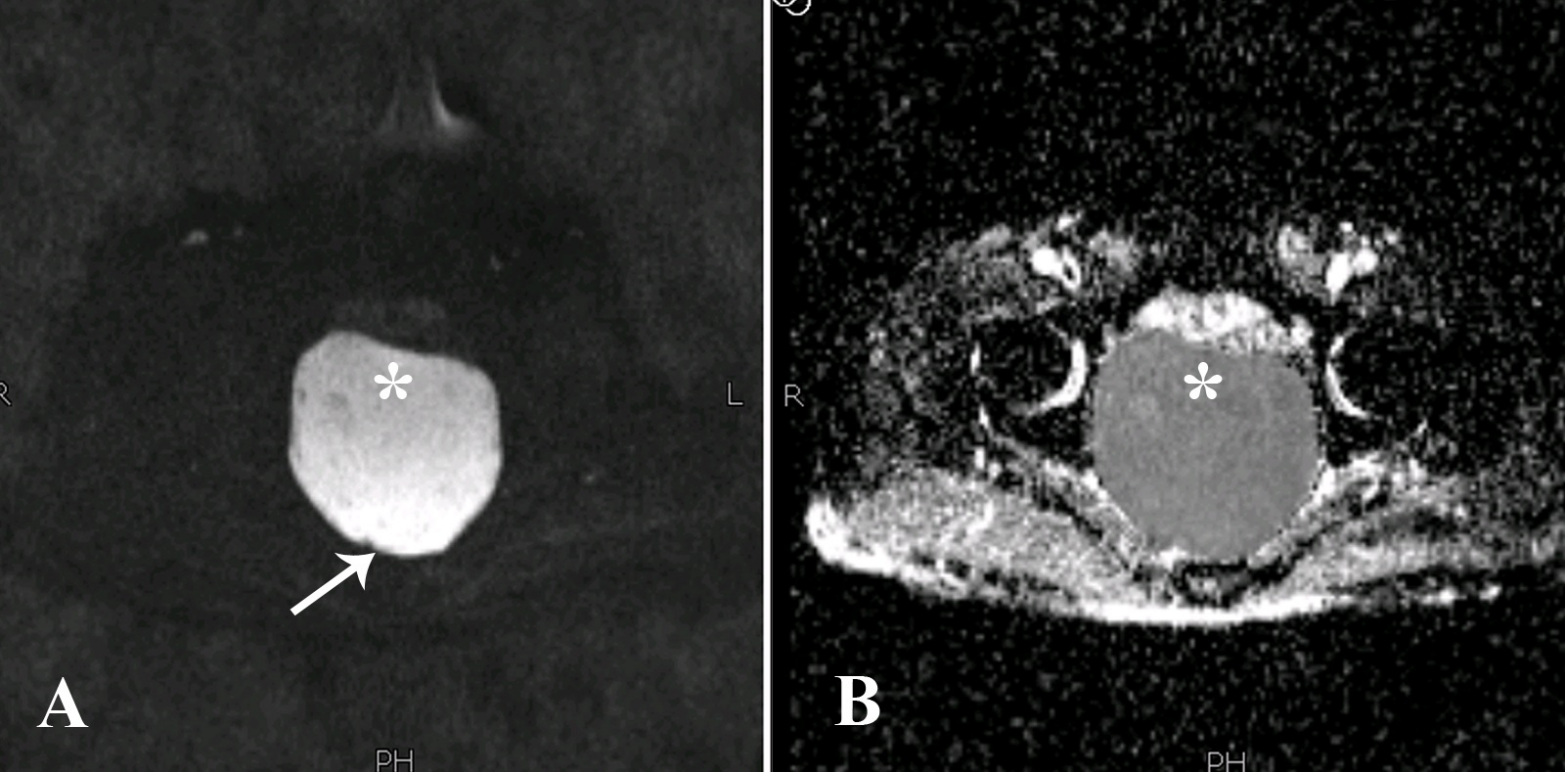
Diffusion weighted sequence (A) and corresponding apparent diffusion coefficient map (B). The lesion (asterisk) was showing diffusion restriction appearing hyperintense on diffusion weighted imaging (A) and showing dropout on the corresponding apparent diffusion coefficient map (B). In addition, there was differential restriction in which posteriorly there were relatively more hyperintense signals on DWI (arrow). DWI: diffusion weighted imaging

The lesion was showing diffusion restriction, appearing hyperintense on diffusion weighted imaging (DWI) and hypointense on the corresponding apparent diffusion coefficient (ADC) map. There was an additional rare feature which we noticed in the diffusion weighted sequences in which differential restriction was seen in its dependent contents (Figure 4).

These MRI features were pathognomonic of an epidermoid cyst. Features did not favor a pelvic abscess, which also shows diffusion restriction on MRI, because the surrounding fat was clear, the adjacent viscera were normal in appearance, and there was no thickened, irregular ring-enhancement of the cyst wall or heterogenous signals within it in any of the sequences. There was also a lack of clinical history of fever, systemic illness, or previous surgery.

**Figure 5:**
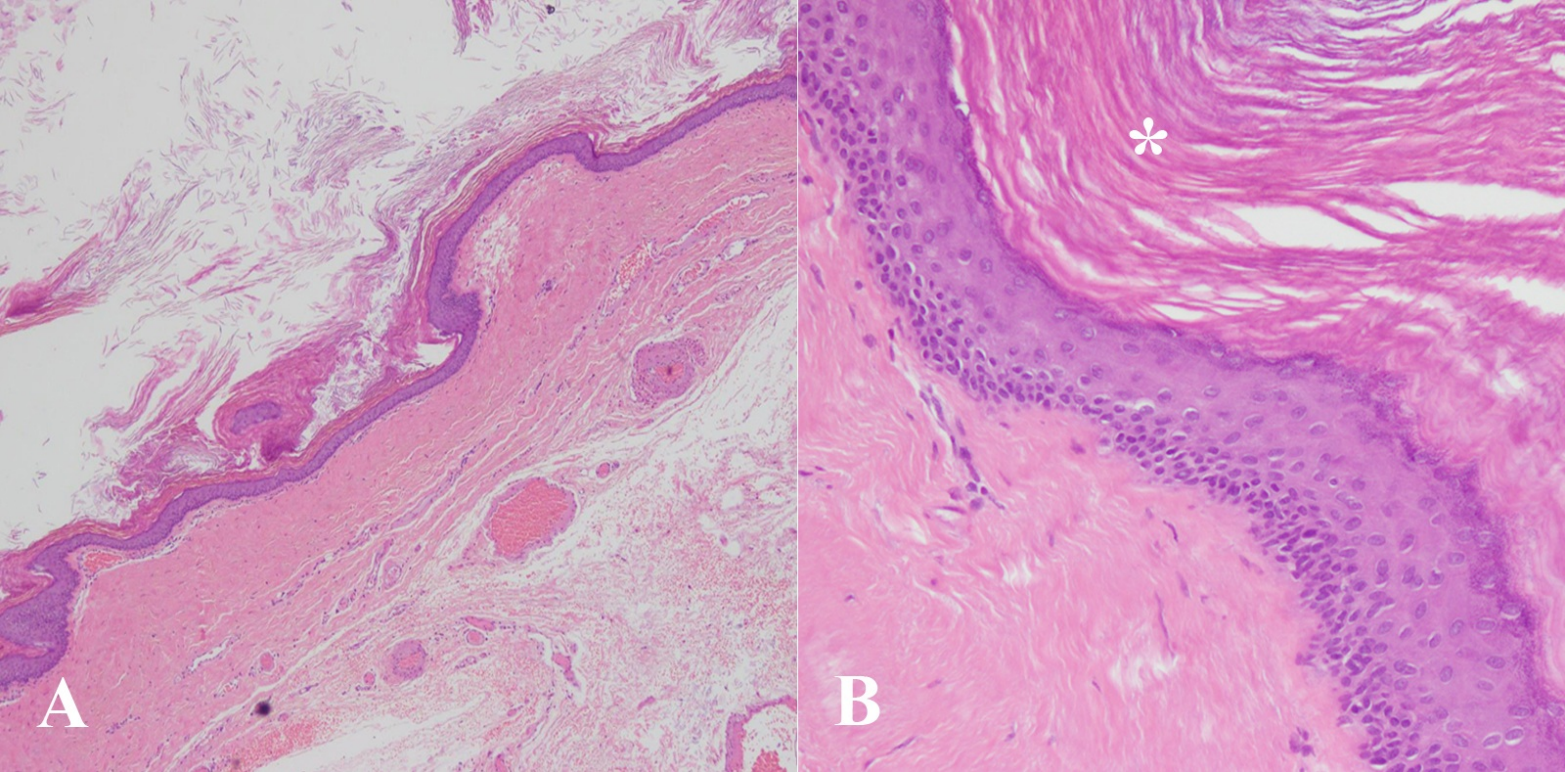
A & B: Histology slides A) Low power view showing cyst wall lined by keratinized squamous epithelium. B) High power view showing cyst wall lined by stratified squamous epithelium with distinct granular cell layer and laminated keratin along luminal surface (asterisk).

The patient subsequently underwent an exploratory laparotomy and complete surgical excision of the cyst with preservation of the adjacent structures. On gross pathological examination, the cyst was filled with keratinous material. Histological sections examined revealed a benign cystic lesion lined by stratified squamous epithelium with keratinization on the luminal surface (Figure 5).

The underlying wall did not show any skin appendages or other teratomatous elements. There was no evidence of malignancy. Overall, the features were in accordance with those of a typical epidermoid cyst. No pus cells were noted to suggest secondary infection.

## Discussion

Developmental cystic lesions arising in the presacral location include a multitude of tumors, including dermoid cysts, epidermoid cysts, chordomas, adrenal rest tumors, anterior sacral meningoceles, cystic hamartomas, tailgut or rectal duplication cysts [6]. Bony defects may be seen in cases of dermoid, tailgut, and neurenteric cysts. In addition, spinal canal or meningeal communication may be demonstrated in anterior sacral meningoceles, while communication with the rectum may be demonstrated in rectal duplication cysts. Tailgut cysts usually show presence of wall calcifications and internal septations. High signals on T1 weighted MR imaging may be seen in tailgut cysts or dermoid cysts due to presence of mucinous material and fat, respectively [1].

Presacral epidermoid cysts, on the other hand, are unique in the sense that they are the only developmental lesions that show diffusion restriction. Previous reports suggest that presacral epidermoid cysts are generally found in females of reproductive age group as incidental findings during gynecologic or obstetric related imaging [2]. Because of their unusual location and slow growth, they have a tendency to remain asymptomatic. Symptoms of pain with presacral lesions have been associated with secondary infection or malignant degeneration [5, 7]. These cysts are generally difficult to characterize on ultrasound and computed tomography. Consequently, definitive preoperative diagnosis is difficult. To solve this diagnostic dilemma, we recommend MRI with the use of diffusion weighted imaging for definite preoperative diagnosis in many lesions. Epidermoid cysts appear as T1 hypointense, T2 hyperintense masses which show diffusion restriction, wherever they are present within the body. T2 hypointense foci may be seen within the lesion because of presence of keratin [2].

A previous study reported that presacral epidermoid cyst was preoperatively diagnosed using endorectal ultrasound, CT and conventional MRI without mentioning diffusion weighted imaging characteristic. They reported that fine needle aspiration was performed for diagnosis [2]. Generally, biopsy or needle aspiration is unwarranted in presacral cystic lesions because of the risk of seeding of malignant cells if present, as well as the potential to secondarily infect the sterile contents of the cyst.

Another recent case report from Japan reported that presacral epidermoid cysts can be characterized using diffusion weighted imaging on MRI of the pelvis [8]. There was an additional rare feature which we noticed in the diffusion weighted sequences, in which there was differential restriction in its contents. Posteriorly, the contents were showing relatively more hyperintense signals on DWI, likely correlating with keratin deposition. This appearance has been reported once before in a presacral epidermoid [8]. MRI may also differentiate between any bony, spinal canal or meningeal involvement, or signs of malignant degeneration [2, 7]. Heterogenous signal intensity on T2, irregular thickened walls, solid component, and presence of enhancement are concerning for malignant change [6].

The closest differential of an epidermoid cyst on basis of diffusion restriction is a retrorectal pyogenic abscess. In such cases, there is often a clinical history of previous surgery, systemic illness or high-grade fever. Additionally, on imaging an abscess is usually ill-defined or loculated with heterogenous signals and presence of a thickened enhancing rim, intracavity fluid debris level or air specks. The surrounding fat may be infiltrated, the rectal wall may be thickened or there may be evidence of diverticulosis depending on the etiology. On DWI, there may be heterogenous diffusion restriction in an abscess which is frequently central. Reports have also suggested that pelvic abscesses generally have lower ADC values than pelvic cystic tumors [9].

Presacral epidermoid cyst is a rare entity, and use of diffusion restriction to characterize them is propitious for preoperative planning. It is recommended that these lesions be surgically excised because of the risk of malignancy or secondary infection. Therefore, correct diagnosis and proper treatment for a presacral lesion is very important as inadequate primary surgery can lead to increased morbidity. It can also increase the risk of recurrence and can even cause complications such as fecal incontinence [4].

## Conclusions

With the advent and availability of higher magnetic strength MRI scanners, the use of diffusion weighted imaging in MRI for pelvic lesion characterization should be performed routinely. Epidermoid cysts in the presacral/retrorectal location are a rare entity which can be accurately diagnosed using diffusion weighted imaging. Therefore, DWI is a useful diagnostic tool in this regard as it obviates the need of biopsy. The characteristic appearance of an epidermoid cyst on MRI with diffusion weighted imaging warrants its use in diagnosis of pelvic cysts and should be made part of the standard protocol for pelvic imaging.
